# Effect of Socio-economic factors on malaria prevalence in a Peri-urban setting in Vihiga County, Western Kenya Highlands

**DOI:** 10.4314/ahs.v24i2.19

**Published:** 2024-06

**Authors:** Beatrice Aleyo Muzame, Elizabeth Omukunda, David Mulama, Patrick Okoth

**Keywords:** Malaria prevalence, socio-economic factors, malaria control strategies, mosquitoes breeding sites, household level

## Abstract

**Background:**

Malaria is the leading cause of mortality in sub-Saharan Africa.

**Objective:**

The study assessed the effect of socio-economic factors on high malaria prevalence in a peri-urban setting in Vihiga County, Western Kenya highlands aimed at strengthening implementation of cost-effective malaria control strategies at household level.

**Method:**

A longitudinal study was carried out in the study area from December 2019 to November 2020. From patients who presented themselves at Mbale Provincial Rural Training health centre for various treatments, 768 malaria confirmed patients were recruited and signed consent before the study commenced. Data was collected using microscopy and structured questionnaires used to stratify malaria patients into socio-economic status and their residence. Data was presented through graphs, frequency, analyzed using linear regression and correlation. P-value ≤ 0.05as considered statistically significant.

**Results:**

Linear regression analysis showed effect of socio-economic factors on malaria prevalence was statistically significant, R2 = 0.061, [F (7,760) = 7.063], p < 0.0001). Level of education, wealth, land size, house type and house ventilation were statistically significant to malaria prevalence as opposed to salary and household size.

**Conclusion:**

Socio-economic factors influenced malaria prevalence in the study area. Implementation of cost-effective malaria control strategies should be strengthened at household level.

## Background Information

Despite numerous global intervention strategies, malaria still remains the leading cause of morbidity and mortality in developing countries such as sub-Sahara Africa^1^, ^2^. A female *Anopheles* mosquito (*Anopheles gambiae, A. arabiensis* or *A. funestus*) takes in human blood meal and when infected with *Plasmodium* parasites, transmit them causing uncomplicated or severe malaria which kills a child every minute globally^3^,^4^. About 99% of malaria in Kenya is *Plasmodium falciparum* malaria while 1% of human malaria infection is caused by *Plasmodium vivax, Plasmodium ovale*, and *Plasmodium malariae*^2^, ^4^, ^5^. Malaria targeted risk populations include; pregnant women, children below five years old, immunosuppressed patients such as those with HIV/AIDS, the aged and travellers to malaria endemic regions due to their low body immunity^6^. In Kenya, universal coverage with malaria prevention is yet to be achieved^4^,^5^. Malaria prevalence in Kenya was 5.08 in 2018, 5.02 million in 2019 and 3.66 million in 2020 with Western Kenya carrying most of the malaria burden where more than 70 % of the population in Western Kenya is at malaria risk^4^, ^5^. Vihiga County where the study area falls is a Lake endemic malaria zone^5^ and 28,786 patients across Vihiga County were diagnosed with malaria between January and March 2019 causing high mortality among children below 5 years old^7^. The study assessed the effect of socio-economic factors on high malaria prevalence in the study area aimed at strengthening implementation of cost-effective malaria control strategies at household level. There are no recorded documents specifically in the study area which have assessed effect of socio-economic factors on malaria prevalence yet socio-economic factors influence malaria prevalence according to ‘a malaria-free Kenya, Kenya Malaria Strategy- 2019-2023’ 5, hence the study.

Socio-economic factors refer to an individual's social and economic status in relation to other people in a given area^4^. A study carried out in Madhya Pradesh, India^8^ and in sub-Saharan Africa^9^, ^10^, ^11^ indicated that malaria burden can be reduced by improving people's socio-economic status. Studies carried out in sub-Saharan Africa^10^, ^11^ and in Assam, India^12^ reported that socio-economic factors that determine malaria transmission by influencing mosquito density^3^ and frequency of mosquito bites included; level of education, house structure, wealth, residence which can be in urban or rural areas, use of bed nets, household size, socio-cultural practices, stagnant water, vegetation, land use, time of fetching water for domestic use, nutrition which was linked to education and economic status and how far homes are from sources of water^4^. Perceptions and knowledge towards malaria infection and antimalarial compliance determines emergence of antimalarial resistance in malaria parasites which is closely associated with wealth and level of education^1^, ^4^. In Nigeria, socio-economic factors influenced malaria incidences in Calabar region^13^ and in Kwara^14^, where treatment and control of malaria attracted more financial and human resources. A study carried out in rural Uganda^15^ and in Chewaka district, Western Ethiopia^16^ indicated that inequalities in socio-economics affected malaria prevalence. Socio-economic status such as wealth, education and salary influences malaria prevalence as it determines the individual's knowledge and attitude towards malaria infection and control leading to malaria health inequalities as it was reported in study findings in lower North, Northern Tanzania^17^, and in Madhya Pradesh, Central India^18^. A malaria indicator survey was carried out in all 47 Counties in Kenya, which associated malaria prevalence with socio-economic factors which included; wealth, household size, house design and education^4^.

Wealth is the main socio-economic factor influencing malaria prevalence because it seems to influence other socio-economic factors^4^, ^5^, ^6^. Wealth in the study area was considered to be the income from various economic activities (type and quantity of crops grown, quality and quantity of livestock kept, fishing, brick making, mining and trading), salary, quantity and quality of assets such as type of the house, vehicle if any, television owned by the head of the household 9. In Assam, India, malaria was considered to be an infectious disease of poverty^12^, and an economic burden disease in Western Ethiopia^16^. Wealth can enable one to purchase enough insecticide-treated mosquito nets for all household members, build houses that can prevent entry of mosquitoes into the house, spray the house regularly with insecticides, buy original antimalarial drugs as counterfeits drugs can cause unavoidable malaria deaths and take his/her household members for malaria treatment early enough^16^, ^18^. A study carried out in Kenya showed that high level of poverty and low education level influenced malaria prevalence^4^, ^5^. Study findings on malaria from other parts of the world^6^ and from Kenya malaria indicator survey4 proved that poverty can led to malaria patients avoiding health facilities but instead engaged in; self-antimalarial prescription without laboratory test for malaria, failed to adhere to antimalarial treatment given, used counterfeit drugs which could have led to antimalarial treatment failure that might have resulted in spread of antimalarial drug resistance in the population that impacted negatively to malaria control. Studies on effect of socio-economic on malaria were carried out in Western Kenya^19^ and in all 47 Counties in Kenya^4^, where study findings of the household malaria survey associated socio-economic inequalities such as wealth (salary included), education level, household size, land size and land use with malaria prevalence^19^. A Study carried out in rural Uganda^20^ associated malaria with poverty.

A study was carried out in Western Kenya highlands on female *Anopheles* mosquitoes and their malaria transmission in regions where bed net ownership was high^21^, but mosquito bed nets are supposed to cover beds yet poverty-stricken individuals lack beds and it becomes difficult for them to effectively use the nets4. In Busia County, Kenya malaria prevalence was high which was linked to socio-cultural practices and economic factors in procurement and utilization of mosquito nets^22^. More studies were carried out on mapping socio-economic inequalities on malaria prevalence in sub-Saharan African countries^23^. Study findings from sub-Saharan Africa on house structure which seem to be linked to economic status and size of the land proved that house structure influenced malaria prevalence^10^. Houses that are close to each other which might be linked to size of the land encourage malaria transmission from one house to another and house designs with ceiling boards and limited ventilation through windows and doors to some extend reduce entry of mosquitoes into the house^2^,^6^,^24^. A multi-country study on housing improvement and malaria risk^10^, and on housing and health^11^ were carried out in sub-Saharan Africa which linked malaria to house design. A study carried out in rice irrigation areas in Western Kenya proved that house designs can reduce the density of female *Anopheles* mosquitoes that rest inside the houses waiting to transmit malaria at dusk influencing malaria prevalence^24^.

Lack of knowledge towards malaria infection and adherence to antimalarial drugs prescribed by health workers which may be linked to wealth and education level was reported in New Guinea and in rural Kenya^25^, ^26^, ^27^. Poverty-stricken population are not exposed to current information through media or through other means such as attending malaria days to be educated on malaria control, drug adherence and compliance according to study findings in Kenya^4^, ^5^, rural Mozambique and other regions in the world and misconception on malaria infection include being rained on or a curse or a punishment from God as opposed to being bitten by mosquitoes which breed in stagnant water^28^,^29^,^30^. Recorded malaria studies in Vihiga County are mainly on effect of climate on malaria prevalence^7^. “A malaria - free Kenya, Kenya Malaria Strategy- 2019-2023” recommended effective malaria prevention strategies by people at risk to reduce economic burden of malaria, yet transmission of malaria is determined by socio-economic factors, hence the study.

## Materials and methods

### Study Site

The study area was Mbale town and its environs which was identified by a global positioning systems (GPS) mapping at Vihiga County headquarters. A baseline survey was done to familiarize with mosquito breeding grounds in the study area before the study commenced. Mbale town is the headquarters of Vihiga County, found within Vihiga and Sabatia sub-Counties, Western Kenya, along Kisumu-Kakamega Road^31^. It is located at latitude 0° 0′54.0′N and longitude 34°43′17.0 E with an altitude of 1200 meters above sea level, rainfall of between 1800mm to 2000mm annually, highest in April (288 mm) and lowest in January 94 mm^31^. Highest average temperature occurs in February (21.4° C) and lowest in July (19.1°C) with an annual average temperature of 20.4° C 7, 31. Economic activities in the study area included; crop farming, livestock keeping, fishing, brick making, mining and trading^7^. This study site was chosen because it had high malaria prevalence and mortality despite various malaria control strategies having been put in place^7^, ^32^. The study area had poverty level of 62% with high dependency ratio of 100.90^33^, which could have contributed towards high malaria prevalence. Population of people in Mbale town and its environs who are mainly Maragoli community was 60,000 according to 2019 population census^33^ and sample size should have been 382 people but the research used 768 people to cover a higher number of malaria patients because the study area is densely populated^34^.

### Study Design

The study adopted a longitudinal study design^35^, which was carried out from December 2019 to November 2020 to cater for both short and long rainy seasons.

### Data Collection

At the triage room of Mbale Provincial Rural Training Health Center, anthropometric characteristics of the study population were recorded. Patients who were observed with signs of malaria by doctors at the health facility used were screened for malaria by qualified laboratory technicians using microscopy and observation as per world health organization standards^28^, while other health workers interviewed malaria patients as they administered semi-structured questionnaires stratifying them into various socio-economic status and their residence as per Kenya Malaria Strategy- 2019-2023 5. Records on malaria prevalence data was obtained from the Mbale Provincial Rural Training Health Center. The area for finger-prick to collect blood was first swabbed with methylated spirit to sterilize it and allowed to dry before blood collection was done. Blood films were dried, stained with giemsa stain, immersed in oil and observed under 100 x microscope objectives to examine malaria parasites.

### Inclusive and Exclusive Criteria

The study was purposive and equal numbers of 192 malaria confirmed patients/guardians who were volunteering to participate in the study were recruited for each of the following age groups; children below 5 years old, children between 5-14 years old, children between 14-18 years old and adults. Malaria patients with; allergy to antimalarial drugs, chronic infections and people who had been on antimalarial treatment within the preceding 2 weeks were excluded from the study. Most pregnant women were reluctant to participate in the study and they were also excluded.

### Sample Size Determination

The sample size formula below was adopted from Krejcie, R.V and Morgan, D.W., 1970 (34).

S = X2NP(1-P)/d2(N-1) +X2P(1-P)

S =Desired sample size

X = Z-value = 1.96 for 95% confidence level, N = Population size (60,000)

P = Population proportion = 0.5,

d = degree of accuracy = 0.05,

S = (1.96)2(60,000) (0.5) (0.5)/(0.05)2(59,999) + (1.96)2(0.5) (0.5)

S = 382.

### Data management and analysis

Data was entered in spread sheets and analysed using a SPSS software version 17. Descriptive and inferential data analyses were done. Graphs and frequency tables were used to show distribution of socio-economic factors in the study population while linear regression and correlation were used to analyse effect of various socio-economic factors on malaria prevalence in the study area. P-value ≤ 0.05 was considered statistically significant in all tests.

### Ethical considerations

Masinde Muliro University of Science and Technology Institutional Ethical Review Committee granted an ethical review letter (MMU/COR: 509099) while National Commission for Science, Technology and Innovation provided License number: NACOSTI/P/20/3379 for data collection. A written informed consent was signed by adult malaria patients and parents or guardians signed for malaria patients below 18 years old before the study commenced and their confidentiality was guaranteed.

## Limitations of the Study

The research was conducted during the COVID-19 pandemic period. Most patients were reluctant to visit health facilities during this period for fear of being infected with COVID-19.

## Results

From linear regression analysis obtained, R2 was 0.061 [F (7,760) = 7.063], p < 0.000) showing that overall socio-economic factors had statistically significant effect on malaria prevalence. Individual socio-economic factors that were statistically significant to malaria infection in the study area were; level of education ([F (7,760) = 7.063], p-value = 0.002), wealth [F (7,760) = 7.063], p-value = 0.000, size of land ([F (7,760) = 7.063], p-value = 0.006, house type ([F (7,760) = 7.063], p-value = 0.000 and ventilation in the house ([F (7,760) = 7.063], p p-value = 0.048 as opposed to salary ([F (7,760) = 7.063], p-value = 0.828) and household size [F (7,760) = 7.063], p = 0.916) as shown in table 1.

**Table 1:** Linear Regression Results Showing Effect of Socio-economic Factors on Malaria Infection in the Study Area

Coefficients					
Model	Unstandardized Coefficients		Standardized Coefficients	T	Sig.
	B	Std. Error	Beta		
Constant)	518.597	68.263		7.597	.000
Level of education	22.966	7.562	.107	3.037	.002
Salary	2.776	12.785	.009	.217	.828
Wealth	-53.833	12.264	-.176	-4.389	.000
Size of land	15.507	5.592	.099	2.773	.006
Household size	-.967	9.156	-.004	-.106	.916
House type	-37.786	10.441	-.152	-3.619	.000
Ventilation in the house	37.454	18.883	-.083	-1.984	.048

**Figure 1:**
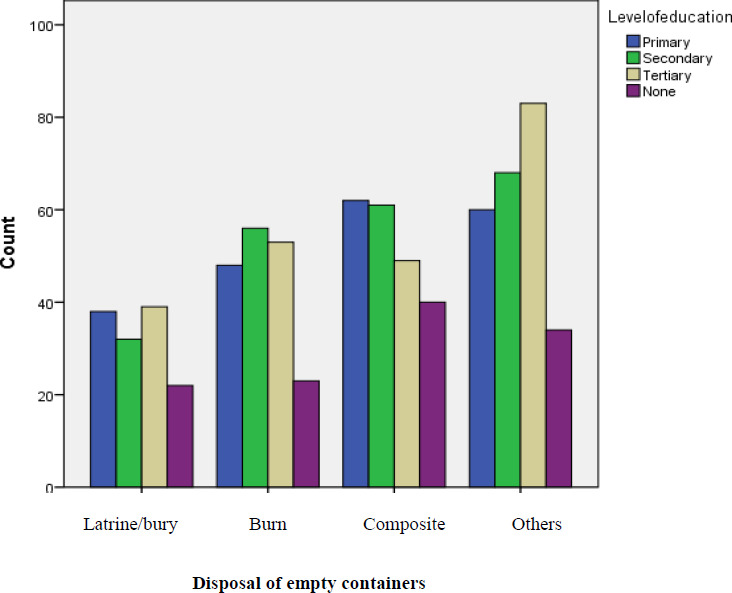
Relationship Between the Head of a Household's Level of Education and Disposal of Empty Containers

From analysis obtained, study participants seemed to have deposited empty containers appropriately which included selling them to be recycled (others) irrespective of their level of education as shown in figure 1 which might have influenced malaria prevalence.

Analysis obtained showed correlation between information on mosquito's breeding grounds was statistically insignificant to education and wealth while correlation between level of education and malaria prevalence and correlation between wealth and malaria prevalence was statistically significant as shown in table 2.

**Table 2:** Correlation of Level of Education, Wealth, Information on Mosquito Breeding Grounds and Malaria prevalence of Study Population in the Study Area

		Malaria	Level of Education	Wealth	Information on mosquito breeding grounds
Malaria	Pearson Correlation Sig.(2-tailed) N	1	.088*	-.158**	.079*
			.014	.000	.028
		768	768	768	768
Level of education	Pearson Correlation Sig.(2-tailed) N	.088*	1	.062	-.010
		.014		.084	.772
		768	768	768	768
Wealth	Pearson Correlation Sig.(2-tailed) N	-.158**	.062	1	.028
		.000	.084		.447
		768	768	768	768
Information on mosquito breeding grounds	Pearson Correlation Sig.(2-tailed) N	.079*	-.010	.028	1
		.028	.772	.447	
		768	768	768	768

* Correlation is significant at the 0.05 level (2-tailed).

** Correlation is significant at the 0.01 level (1-tailed).

**Figure 2:**
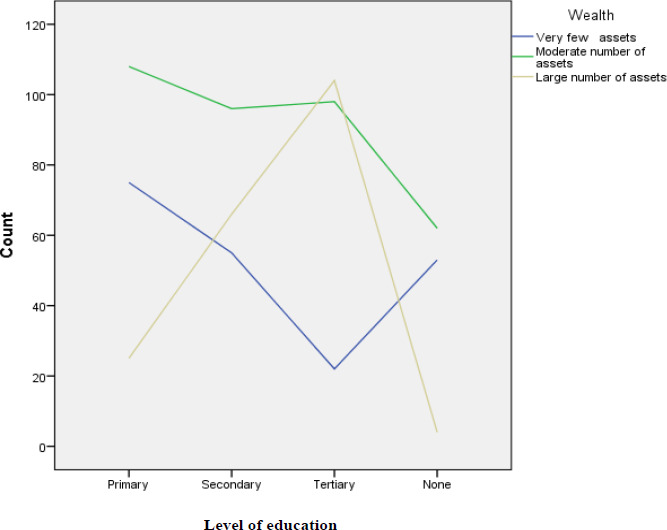
Relationship Between the Head of a Household's Wealth and Level of Education in the Study Area

Wealth in the study referred to quality and quantity of assets one owned which included sources of income such as salary, vehicles, livestock, farms, rental houses, businesses. Televisions, electricity, telephones, and radios are very important in delivering malaria prevention programs. From analysis obtained, it was observed that, large numbers of assets were owned by study participants who had high level of education (tertiary) as shown in figure 2.

Distribution of salary among study population in the study area was high among individuals earning between Ksh.10, 000 to Ksh. 20,000 per month (51.4 %) who were considered to be earning moderate salary, followed by those who were considered to have had low salary of below Ksh. 10,000 per month (27.9 %), while those earning high salary of over Ksh. 20,000 per month accounted for 20.7 % (Table 3). Most homesteads recorded household size of more than 4 members (57 %) as shown in table 3.

**Table 3:** Salary Distribution of Head of Household and Household Size in the Study Area

	Frequency	Valid Percent	Cumulative Percent
**Salary**			
High	159	20.7	20.7
Moderate	395	51.4	72.1
Low	214	27.9	100
**Total**	**768**	**100**	
**Household size**			
Two	36	4.7	4.7
Three	86	11.2	15.9
Four	208	27.1	43
More than four	438	57	100
**Total**	**768**	**100**	

**Salary:** Low below Ksh. 10,000/month, Moderate Ksh. 10,000-20,000/month, High over Ksh. 20,000/month).

Most participants in the study area lived in areas where the land size was less than 2 acres (92 %). The land which was more than 2 acres in the study area accounted for only 7.9 % (Table 4).

**Table 4:** Size of the Land for Homesteads and the Head of Household's Level of Education in the Study Area

	Frequency	Valid Percent	Cumulative Percent
**Size of the land**			
Less than 1 acre	237	30.9	30.9
1- 1.5 acres	226	29.4	60.3
1.5 – 2 acres	120	15.6	75.9
More than 2 acres	61	7.9	83.9
Rented houses on	124	16.1	100
less than one acre			
**Total**	**768**	100	
**Level of Education**			
Primary	208	27.1	27.1
Secondary	217	28.2	55.4
Tertiary	224	29.2	84.5
No formal education	119	15.5	100
**Total**	**768**	**100**	

Study population who recorded tertiary level of education constituted 29.2 %, secondary education recorded 28.2 %, primary level of education constituted 27.1 % while 15.5 % of the study population did not have formal education (Table 4). However not all of the study population in the study area who recorded tertiary education were employed.

Permanent houses in the study area constituted 42.6 % while other types of houses constituted 57.4 % (Table 5).

**Table 5:** Types of Houses in the Study Area

House Type	Frequency	Valid Percent	Cumulative Percent
Permanent (cemented floor/bricks/stone wall/iron/tile roof with ceiling boards)	327	42.6	42.6
Semi-permanent (mud floor mud walls/iron roofed	309	40.2	82.8
Semi-permanent (cemented floor/mud walls/iron roofed	73	9.5	92.3
Others (iron walls/roof/cemented floor)	59	7.7	100
**Total**	**768**	**100**	

**Figure 3:**
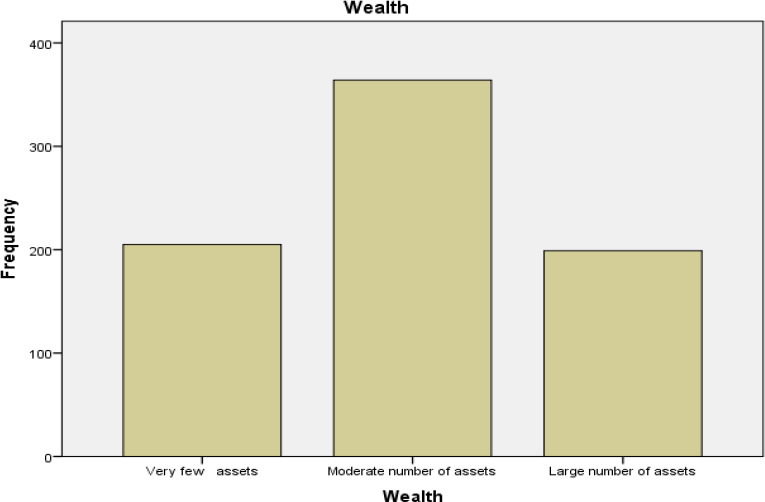
A Bar Graph Showing Wealth in among Study Population Study in the Study Area

Most individuals in the study area had moderate number of assets which accounted for 47.4 %. Individuals who recorded large number of assets accounted for 25.9 % while individuals with very few assets accounted for 26.7 %. as shown in figure 3.

## Discussion

Socio-economic factors in the study were considered to be risk factors in malaria prevalence. Socio-economic factors that were considered in the study area included; level of education, salary, wealth, size of the land, household size, types of houses and ventilation in houses. Overall socio-economic factors in the study area were statistically significant (p-value 0.00) to malaria prevalence although the household size (p- value = 0.916) and salary of the head of the household (p- value = 0.828) were statistically insignificant. The following are the individual socio-economic factors that showed statistically significant effect on malaria prevalence; wealth (p- value = 0.000), size of land (p- value = 0.006), house type (p- value = 0.000), other sources of ventilation in the house besides lack of ceiling board (p- value = 0.048) and level of education (p- value = 0.002). From the study area, level of education did not affect disposal of empty containers. From analysis obtained, it was observed that large numbers of assets (57%) were owned by participants who had high level of education (tertiary education which accounted for 29.2%) which might have reduced malaria prevalence in such individuals. Knowledge on stagnant water as breeding ground of mosquitoes plays a vital role in malaria prevention. Analysis obtained showed correlation between information on mosquito's breeding grounds was statistically insignificant to education and wealth while correlation between level of education and malaria prevalence and correlation between wealth and malaria prevalence was statistically significant.

Most heads of the household recorded salary of Ksh. 10,000 to Ksh. 20,000 per month while those earning high salary of above Ksh. 20,000 per month accounted for 20.7 % which might have compromised malaria prevalence in the study area. Most homesteads in the study area occupied less than 2 acres of land (75.9%) which might have encouraged transmission of malaria from one house to another. Most study participants who were more than four (57%) in a homestead and those who also earned low income experienced challenges in purchasing adequate mosquito prevention bed nets which might have exposed them to mosquito bites, increasing malaria prevalence. Houses without ceiling boards and with more ventilation through windows and doors in the study area which accounted for 57.4% to some extend allowed entry of mosquitoes into the house which increased malaria prevalence. The Kenyan government should consider improving socio-economic status of poor population and target malaria control at household level in order to reduce malaria prevalence as recommendations by Degarege^9^ and attain a malaria free Kenya, Kenya Malaria Strategy- 2019-2023 5.

Socio-economic factors were considered to be risk factors in malaria transmission which influenced malaria prevalence^3^, ^9^, ^36^. Socio-economic factors in the study area were statistically significant (p-value 0.00) to malaria prevalence supporting study findings in Western Kenya Highlands^19^, Busia^22^ and Siaya^19^ Counties in Kenya, Tanzania^17^, sub-Sahara African countries^12^, ^23^, India^8^, Pakistan^36^, Nigeria^13^, ^14^, and Ethiopia^16^. High level of education in the study area seemed to have enabled individuals to understand the causes, management and prevention of malaria as opposed to individuals with low or no formal education, supporting study findings in rural Uganda^15^,^20^ and in endemic primary health centres in Assam, India^12^. From the results obtained in the study area, a good number of study participants who either dropped at primary school level and those without any formal education in study area tended to link malaria to feeding on first harvest of maize and beans, curse from God and being rained on supporting study findings from Kenya Malaria Indicator Survey, 2020 4 on malaria beliefs. In some cases, study population seemed to have preferred white bed nets to the green or blue nets which they claimed suffocated them and led to miscarriage in pregnant mothers. This misconception led them to use mosquito nets to cover their fish and vegetables thus increasing malaria prevalence in the study area. Most study participants with no formal education also found it difficult to link malaria to stagnant water and mosquito bites.

Wealth in the study was considered to be the income from the salary if employed and quantity and quality of assets owned by the head of the household which was statistically significant to malaria prevalence. One can be wealthy without necessarily being well educated. Wealth affected malaria prevalence in study area supporting study findings that were carried out in rural Uganda^20^, which linked malaria to poverty. Most individuals who earned over 20,000ksh per month (25.9%) in the study area (availability of money and property in this case) were able to purchase enough insecticide-treated mosquito nets for all household members in consistence with findings from Western Kenya highlands^21^, built houses with ceiling board to prevent entry of mosquitoes into the houses supporting findings in other regions in Western Kenya^24^ and sub-Saharan Africa^10^, sprayed houses regularly with insecticides, bought original antimalarial drugs as counterfeits drugs can cause unavoidable malaria deaths and availability of money also enabled individuals to take their household members for malaria treatment early enough^4^, ^5^. However, level of education and wealth of the study population were statistically insignificant to information on breeding grounds of mosquitoes which transmitted malaria. Poverty level in study area was very high (62%) with high dependency level^31^, ^33^, which was a challenge to participants with low salary and very few assets to effectively engage in quality malaria management, similar to findings in Busia County, Kenya^22^, rural Uganda^20^, Chewaka district, Western Ethiopia^16^, and in sub-Saharan Africa countries, which linked malaria to poverty. One can be well educated with a lot of information on how to treat and prevent malaria but may lack money may be due to being unemployment which may prevent a person from controlling or treating malaria effectively. From correlation analysis obtained in the study area, wealth was statistically insignificant to level of education and information on mosquito breeding grounds.

The size of the land to some extend determined malaria transmission which influenced malaria prevalence where individuals with comparatively large pieces of land over 2 acres in the study area were able to farm far from the location of their houses as opposed to close houses which were observed in areas where the sizes of land were less than 2 acres. Mosquitoes easily transferred malaria parasites from one house to another in compounds where the sizes of land were small which increased malaria prevalence in the study area. In cases where farmers decided to construct fish ponds for their income generation on small pieces of land, the stagnant water in the fish ponds attracted breeding of mosquitoes increasing malaria prevalence. Research participants who were more than 4 in a home with low income in the study area were unable to buy adequate mosquito prevention bed nets to increase the number the Kenyan government provided which exposed them to more mosquito bites increasing malaria prevalence. Participants from low income homesteads in the study area used insecticide-treated bed nets in turns while some decided to tear them to enable everybody in the home to tie the net on their heads at night to prevent mosquito bites which increased malaria prevalence in the study area.

House designs in some parts of the study area lacked ceiling boards (57.2 %) which influenced entry of mosquitoes into the houses supporting study findings that were carried out in rice irrigation areas in Western Kenya^24^ and in sub–Sahara Africa^10^, ^11^. Number of windows and doors in a house which were used as house ventilation created entry of mosquitoes into the house and hence increasing malaria prevalence. During the study, it was observed that participants in some homesteads lacked vital information on mosquito breeding grounds. They did not associate fish ponds, disposal of empty containers, creating burrow pits by gold mining and brick making with malaria. In some cases, participants failed to adhere to, and comply with antimalarial drugs and their knowledge on causes and prevention of malaria was poor supporting a study that was carried out in Papua New Guinea and other parts of the world^25^, ^27^,^30^. In Nigeria, socio-economic factors influenced malaria incidences in Calabar region where treatment and control of malaria attracted more financial and human resources in the budget of Nigeria government^13^. The Kenya government should emulate malaria case management from Nigeria by campaigning for more malaria treatment and control funding from other donor countries to increase the 26% yearly budgetary allocation on health which malaria treatment and control takes most of it^4^. The African Union agenda 2063 include; quality of life and well-being, health and economic growth^37^ and health as one of Kenya's big four vision 2030 38 aim at reducing poverty and improving health which should in turn decrease malaria burden in Kenya and other African countries.

## Conclusion and recommendations

Socio-economic factors influenced malaria prevalence in the study area as they determined malaria transmission. Malaria prevalence was high among individuals from poverty-stricken homesteads. Malaria control strategies should be strengthened in study area and in Kenya at household level to reduce malaria prevalence. The Kenya Malaria Strategy- 2019-2023 objective to achieve a malaria-free Kenya can be realized by strengthening implementation of malaria control strategies which is linked to socio-economic factors. There should be appropriate economic and social interventions in low- income and poverty- stricken individuals in the study area, Kenya and the whole world to reduce and eliminate malaria burden. Campaign on use of malaria vaccines should be monitored regularly as a way of eliminating spread of malaria transmission especially in rural areas where most low income people who may not have access to internet, newspapers and television live. The Kenyan government should regularly educate people on net treatment and proper use because individuals especially in poverty-stricken areas used torn nets which allowed mosquito bites while others did not use given nets at all which increased malaria prevalence in some regions in the study area. The government should provide free malaria treatment in public health facilities where most poor individuals get their treatment and allow more health personnel to reach remote areas to educate people on malaria treatment and control. The Kenya government and the world at large should also put more emphasis on use of modern technology and laboratories to eliminate circulating counterfeit drugs from the market in order to prevent increase in malaria deaths.

## Supplementary materials

Dataset generated for analysis is availability from the corresponding author.
